# Diagnostic Certainty in Registry-Based Estimates of Innate and Intrinsic Immunity Defects

**DOI:** 10.20411/pai.v11i2.1108

**Published:** 2026-10-05

**Authors:** Nezihe Köker Özer, Ercan Yılmaz, Erdem Topal

**Affiliations:** 1 Inonu University, Faculty of Medicine, Department of Pediatrics, Malatya Türkiye; 2 Inonu University, Faculty of Medicine, Pediatric Allergy and Immunology Department, Malatya, Türkiye

This Letter refers to the article available at doi: 10.20411/pai.v10i2.845

## TO THE EDITOR,

We read with interest the study by Refaat et al describing defects of innate and intrinsic immunity in the Moroccan inborn errors of immunity (IEI) registry [1]. The cohort is particularly valuable because these disorders may remain unrecognized when routine immunological testing is unrevealing. We believe, however, that clarification of diagnostic certainty would strengthen interpretation of the reported frequency and genetic spectrum.

Of 884 patients with IEI, 79 (approximately 9%) were classified as having defects of innate or intrinsic immunity, whereas only 46 (58%) received a genetic diagnosis [1]. The Methods further state that both genetically confirmed patients and those with clinical or biological findings “suggestive of the condition” were included. The operational criteria used to classify the remaining 33 genetically unresolved patients are not provided. This distinction is important because immunoglobulin concentrations and lymphocyte subsets were largely normal in the cohort, and disorders in this category may require pathway-specific functional testing and/or molecular evidence. Without separating confirmed, probable, and possible diagnoses, the reported 9% may represent a mixture of diagnostic certainty levels rather than the frequency of confirmed innate or intrinsic IEI.

Diagnostic certainty also matters within the genetically resolved group. Table 5 lists variants in *STAT1, IFNGR1, IFIH1, POLR3A, SNORA31,* and other genes, but variant-level ACMG/AMP classification, segregation results, and patient-specific functional evidence are not reported [1]. American College of Medical Genetics and Genomics (ACMG)/Association for Molecular Pathology (AMP) recommendations incorporate population, computational, functional, and segregation evidence when assigning variant pathogenicity [2].

*STAT1* provides a clinically relevant example. In the cohort, *STAT1* variants are assigned to both Mendelian susceptibility to mycobacterial disease and chronic mucocutaneous candidiasis [1]. Yet loss-of-function and gain-of-function *STAT1* variants produce different biological and clinical phenotypes [3, 4]. This distinction can affect surveillance and treatment, including consideration of JAK inhibition in selected patients with *STAT1* gain-of-function disease [4]. Thus, functional direction is not merely descriptive; it can influence clinical management. The 2024 International Union of Immunological Societies (IUIS) classification likewise distinguishes disorders caused by different functional effects of variants within the same gene [5].

We therefore suggest reporting the diagnostic criteria and functional evidence used for the 33 genetically unresolved patients and, for genetically resolved cases, the pathogenicity classification, segregation status, and functional validation of each variant. Stratifying the cohort by diagnostic certainty would preserve the value of this registry while making the 9% estimate more reproducible and clinically interpretable.
